# Impact of fosaprepitant in the prevention of nausea and emesis in head and neck cancer patients undergoing cisplatin-based chemoradiation: a pilot prospective study and a review of literature

**DOI:** 10.1007/s11547-024-01757-3

**Published:** 2024-02-14

**Authors:** Carlotta Becherini, Viola Salvestrini, Isacco Desideri, Giulia Vagnoni, Ilaria Bonaparte, Niccolò Bertini, Chiara Mattioli, Lucia Angelini, Luca Visani, Vieri Scotti, Lorenzo Livi, Saverio Caini, Pierluigi Bonomo

**Affiliations:** 1 Radiation Oncology Unit, Azienda Ospedaliero-Universitaria Careggi, University of Florence, Florence, Italy; 2 Clinical Epidemiology Unit, Institute for Cancer Research, Prevention and Clinical Network (ISPRO), Florence, Italy; 3 Cancer Risk Factors and Lifestyle Epidemiology Unit, Institute for Cancer Research, Prevention and Clinical Network (ISPRO), Florence, Italy

**Keywords:** Fosaprepitant, Chemotherapy-induced nausea, Emesis, Chemoradiotherapy, Head and neck cancer

## Abstract

**Purpose:**

Cisplatin-based chemoradiotherapy (CRT) is standard treatment for head and neck squamous cell carcinoma (HNSCC). However, IMRT may increase chemotherapy-induced nausea and vomiting (CINV). The purpose of this study is to investigate the effect of fosaprepitant in preventing CINV.

**Methods:**

An infusion of 150 mg fosaprepitant was given through a 30 min. We assessed acute toxicity using CTCAE v.4 and the incidence of CINV using the FLIE questionnaire. The evaluation of CINV was done at the second and fifth weeks of CRT and 1 week after the end. The EORTC QLQ-HN 43 questionnaire was administered before treatment beginning (baseline), at second (T1) and fifth (T2) weeks. A dosimetric analysis was performed on dorsal nucleus of vagus (DVC) and area postrema (AP).

**Results:**

Between March and November 2020, 24 patients were enrolled. No correlation was found between nausea and DVC mean dose (*p* = 0.573), and AP mean dose (*p* = 0.869). Based on the FLIE questionnaire, patients reported a mean score of 30.5 for nausea and 30 for vomiting during week 2 and 29.8 for nausea and 29.2 for vomiting during week 5. After treatment ended, the mean scores were 27.4 for nausea and 27.7 for vomiting. All patients completed the EORTC QLQ-HN 43. Significantly higher scores at T2 assessment than baseline were observed.

**Conclusions:**

The use of fosaprepitant in preventing CINV reduced incidence of moderate to severe nausea and vomiting. No correlation has been found between nausea and median dose to DVC and AP.

**Supplementary Information:**

The online version contains supplementary material available at 10.1007/s11547-024-01757-3.

## Background

Currently, level 1 evidence supports cisplatin-based chemoradiotherapy (CRT) as standard of care treatment [1] in the curative setting of locally advanced head and neck squamous cell carcinoma (HNSCC). The association of cisplatin (CDDP) to radiation (RT) yields a better survival outcome than RT alone, partially offset by the frequent development of prohibitive toxicity [2–4]. High emetogenic chemotherapy (HEC) is defined as the occurrence of emesis in more than 90% of patients without any preventive measures [5]. Since its clinical introduction back in the 1960s, the emetogenicity of CDDP has been recognized [6] as an extremely disturbing side effect, through both vagal peripheral and central mechanisms [7].

Chemotherapy-induced nausea and vomiting (CINV) and radiotherapy-induced nausea and vomiting (RINV) are extremely discomforting complication of oncological treatment which negatively impact on the cancer patients’ QoL resulting in emotional distress, loss of appetite and interference with the activities of daily living [8–10]. However, few data are available on how patients’ quality of quality of life (QoL) is specifically affected by nausea and emesis during CRT. Notwithstanding the adoption of preventive antiemetic regimens, approximately 60–80% and 50–80% of patients experienced nausea or vomiting caused by chemotherapy [11] and by radiotherapy [12], respectively.

In the past two decades, significant advancements have been made regarding the prevention of CINV. In 2019 Razvi et al. published the comparison of the latest antiemetic guidelines (ASCO, NCCN and MASCC/ESMO) [13] recommending the adoption of a triple combination consisting of anti-5-hydroxytryptamine type 3 (5-HT3) receptor, dexamethasone and anti-neurokinin1 (NK) receptor for HEC regimens such as CDDP-based chemotherapy.

The first NK receptor antagonist (RA) to be approved for CINV therapy was aprepitant. Due to the poor water solubility of aprepitant, it was available only in the oral formulation with consequent risk of poor compliance in patients who were unable to receive oral administration. Conversely, the NK RA fosaprepitant (FOS) is a pro-drug of aprepitant which can be conveniently administered through a single fixed dose ev infusion. A large randomized study [14] on a heterogeneous population of patients undergoing HEC regimens demonstrated the efficacy of FOS over placebo, if added to a serotonin–steroid combination, in significantly increasing the frequency of complete absence of vomiting and need of additional rescue medications. Furthermore, in HNSCC patients undergoing CRT, the prevention of acute and delayed CINV is extremely relevant in light of treatment-related toxicity (mucositis, sticky saliva, dysgeusia) and symptoms induced by the disease itself, such as pain and dysphagia, ultimately resulting in weight loss and markedly impaired nutrition status. The activity of FOS—in preventing nausea and emesis—in patients undergoing CDDP-based CRT for HNSCC is only sparsely addressed in the literature. Due to the insufficient available evidence, the undertreatment of CINV and RINV remains a crucial factor for the management of HNSCC patients.

Additionally, in daily clinical practice there is still the relevant need to increase the guidelines adherence rate to enhance CINV and RINV control strategies for patients receiving HEC with or without RT [15]. In the last 2 years, the adoption of FOS into a triple combination for HEC treatments was implemented in our clinic. The aim of our work is to report the efficacy and safety of a FOS-based regimen in a prospective, pilot single-center experience of HNSCC patients undergoing standard CRT. In addition, we investigated whether the occurrence of CINV had a significant impact on patients’ QoL. Moreover, we reported the correlation between dosimetric parameters for vomiting structures and RINV in order to investigate the impact of RT on CINV.

## Material and methods

### Patients and treatment characteristics

Patients affected by histologically proven locally advanced HNSCC candidates to CRT were eligible in our single-center, prospective study. Upon inclusion, performance status (PS) was 0–1 according to Eastern cooperative oncology group (ECOG) score and disease stage was AJCC/TNM III-IV (7th edition). Concurrent CRT was administered either as definitive treatment for primary tumor of oropharynx, larynx and nasopharynx or as adjuvant therapy for operated oral cavity and oropharynx cases with pathologic high-risk features (positive resection margins and/or extracapsular nodal extension). The Charlson comorbidity index (CCI) was employed to assess the cumulative presence of comorbidities at the time of HNSSC diagnosis [16]. History of tobacco exposure was described in terms of packs/year. A computed tomography (CT) scan (Light Speed 16; GE Healthcare Medical Systems, Milwaukee, WI, USA) with a 3 mm slice thickness was obtained for RT planning purpose. All patients underwent the creation of a personalized thermoplastic mask. Intensity modulated radiotherapy (IMRT) was adopted to deliver a total dose of 66–69.9 Gy, with 2–2.12 Gy per fraction in 33 fractions over 7 weeks for adjuvant or definitive cases, respectively. Cisplatin was administered concurrently, with a dose of 100 mg/m^2^ every 3 weeks or 40 mg/m^2^ weekly, as determined by the physician based on the patients’ ECOG PS (0 vs 1) and age-adjusted CCI (< or > 3). The target cumulative dose of cisplatin was 200 mg/m^2^. According to MASCC-ESMO guidelines, antiemetic treatment was administered at cisplatin delivery as follows: Ondansentron 8 mg in 100 ml, dexamethasone 12 mg in 100 ml and FOS 150 mg in 250 ml 30 min before cisplatin infusion on day 1, followed by oral dexamethasone 8 mg on days 2–4. In case of persistent nausea or vomiting, rescue medications were metoclopramide 10 mg and ondansetron 8 mg vials. Before CRT started, an individualized nutritional counseling was scheduled for all patients. All participants included in the study provided informed consent and consent for data processing.

### Outcome measures

Acute toxicity was assessed on a weekly basis and reported according to CTCAE v.4.01. By definition, CINV was graded according to CTCAE “nausea” and “vomiting” items. The absence of significant nausea was defined as G0–G1 scores combined. Complete response (CR) was defined as no emesis and no need of additional medications throughout the course of CRT. The study reported on the tolerability of RT and chemotherapy based on two measures: the mean number of interruptions during RT and the mean cisplatin relative dose intensity during chemotherapy. Additionally, the extent of weight loss at the end of CRT was measured using both absolute values (less than 5 kgs, between 5 and 10 kgs, more than 10 kgs) and percentage values (less than 5%, between 5 and 10%, more than 10%). To investigate CINV impact on patients’ QoL, the Functional Living Index-Emesis (FLIE) questionnaire [17, 18] was administered at baseline (day before CRT start) and at day 1 of weeks 2 and 5, considering the most common timing of CINV onset. FLIE is a patient-reported outcome measure (PROM) consisting of 18 items with a 7-point scale addressing the multidimensional relevance of nausea and vomiting (9 items each). Individual scores were transposed to a 100-point scale, with higher scores corresponding to better QoL. To investigate how CINV may influence head and neck cancer-specific QoL on a broader scope, the EORTC QLQ-HN 43 was administered in parallel to the FLIE questionnaire [17]. Two anatomic structures located in the brain stem, the area postrema (AP) and dorsal vagal complex (DVC) are putatively considered to constitute the vomiting center. Exploratively, AP and DVC were identified on planning CT’s and contoured, following literature indications [19]. IMRT planning was performed without any attempt to avoid the vomiting center. The mean dose received by AP and DVS was recorded for each patient.

### Statistical analysis

Descriptive statistics were utilized to present various features related to CINV, patient (PS, smoking status, CCI), disease (primary site, stage) and treatment (tolerability to RT and cisplatin, weight loss)—as mean and median values with a range for continuous variables and as proportions for categorical variables. To determine any correlations between the development of nausea and significant nausea with patient, disease and treatment characteristics, *t*-test and Mann–Whitney test were employed for continuous parametric and nonparametric variables, respectively, while Chi-square test was utilized for categorical variables. A *p*-value of ≤ 0.05 was considered statistically significant. When multiple risk factors with a *p*-value < 0.05 were identified in the univariate analysis, a multivariate Cox regression analysis was conducted. The differences in mean FLIE were analyzed using a Wilcoxon test. We utilized mixed linear regression models to examine changes over time in the average values of the HN43 multi-item scores and their relationship with patient, tumor and treatment characteristics while accounting for within-person correlation.

## Results

**Fig. 1:**
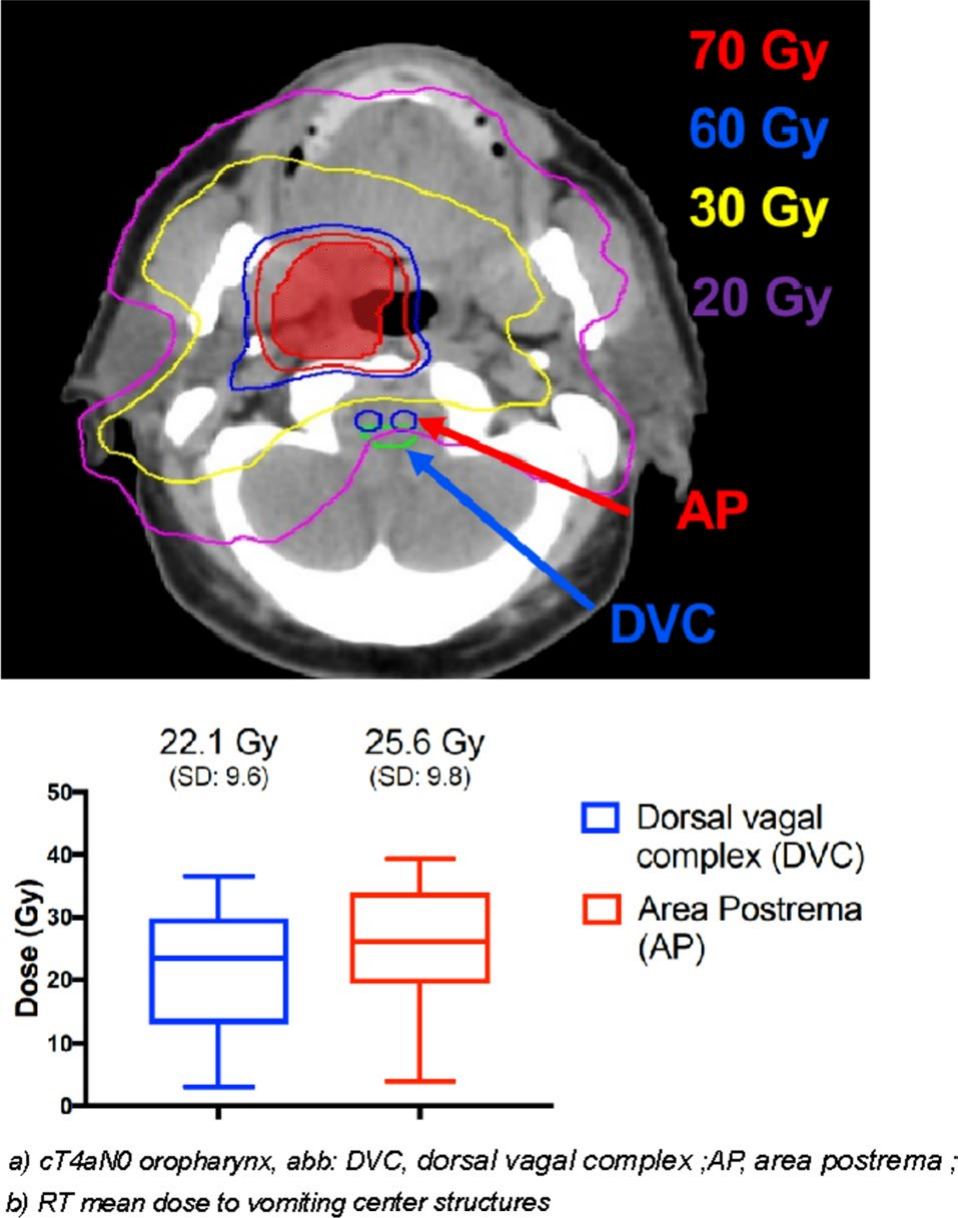
Dose distribution and dosimetric data for dorsal vagal complex (DVC) and area postrema (AP). **a** cT4aN0 oropharynx, DVC, dorsal vagal complex; AP, area postrema; **b** RT mean dose to vomiting center structures

**Fig. 2:**
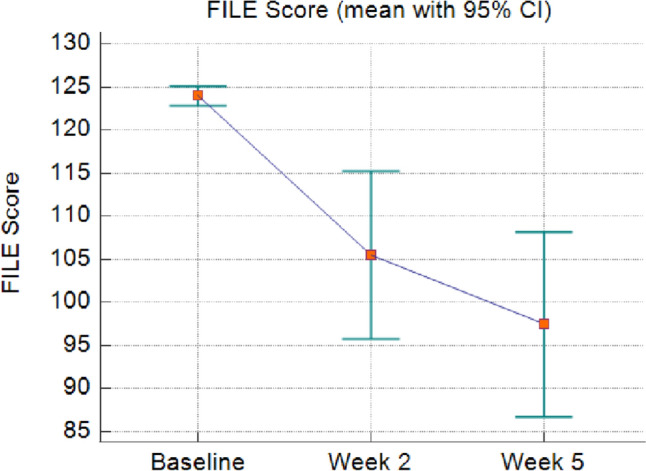
FLIE score assessment at baseline, after 2 and 5 weeks

**Fig. 3:**
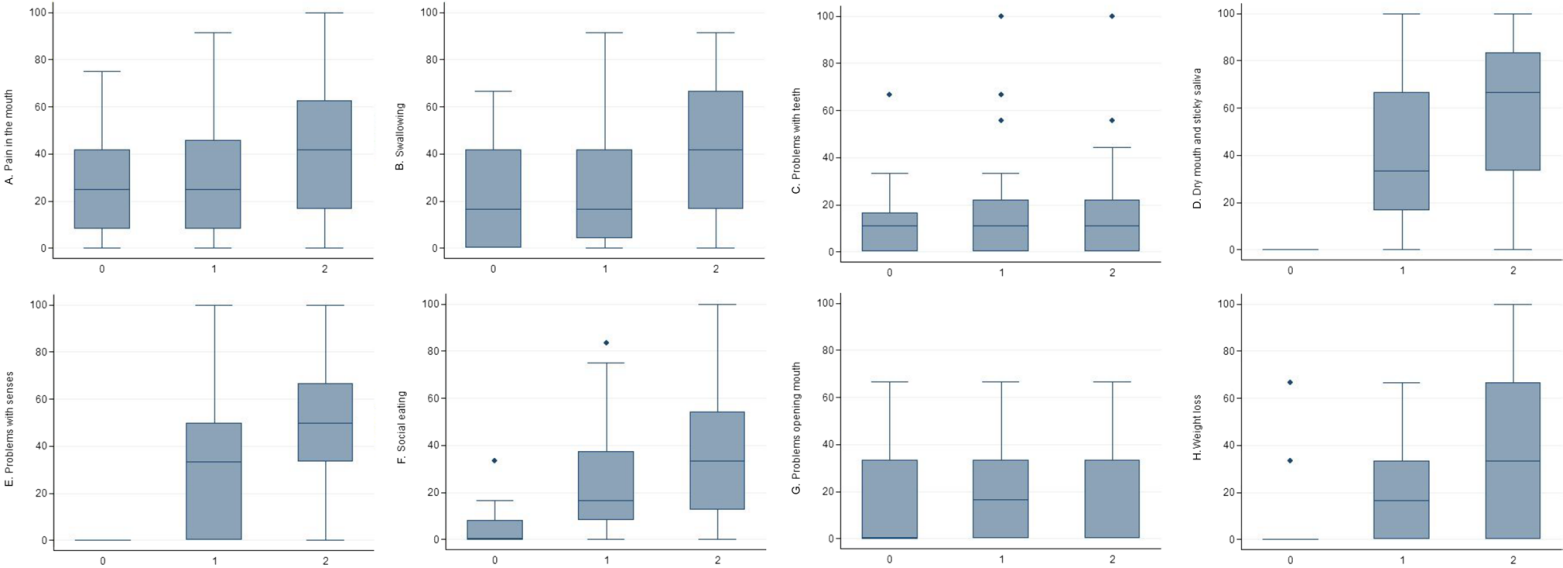
Eating-related domains (**A**. pain in the mouth, **B**. swallowing; **C**. problems with teeth; **D**. dry mouth and sticky saliva; **E**. problems with senses; **F**. social eating; **G**. problems opening mouth; **H**. weight loss) of HN43 questionnaire at baseline, T1 and T2 assessment

Between January 2020 and December 2020, a total of 24 patients (19 males and 5 females), with a median age of 64 years at the time of diagnosis, were enrolled in our study. Less than half of them (37.5%) had a heavy smoking history, defined as a total pack-year (p/y) score greater than 20. All patients had a histologically confirmed diagnosis of head and neck cancer: 14 (58.4%) were affected by oropharyngeal primary tumors, 3 patients each (12.5%) by laryngeal and oral cavity tumors, 2 (8.3%) patients had a nasopharyngeal carcinoma, while primary tumor site remained unknown in 2 of cases (8.3%). Analysis of HPV status was performed in 17 patients: 23.5% (*n* = 4) of them resulted in HPV negative, while 76.5% (*n* = 13) HPV-positive. Patients’ comorbidity was evaluated using Charlson Comorbidity Index and staging was determined according to the AJCC/TNM VII edition. Baseline population characteristics are reported in Table 1. Chemoradiation was administered with disease-curative intent. RT was administered between July 2018 and October 2019, with the use of helicoidal-IMRT with simultaneous integrated boost (SIB) for all patients. The mean total dose to PTV1, PTV2 and PTV3 was 69.3, 59 and 52.8 delivered in fractions of 2.12, 1.8 and 1.6 Gy, respectively. Nineteen of the 24 patients were treated with 3 PTV dose levels. Data regarding doses to the AP and DVC were available for 19 patients. Mean dose to AP was 23.07 Gy (SD +/− 11.58) and 21 Gy to the DVC (range 3.4–39.4 Gy). Mean AP volume was 0.18 cc (SD +/− 0.14) while DVC volume was 0.78 cc (SD +/− 0.6) (Fig. 1). Only 4 patients had to interrupt RT for acute toxicity (mainly dysphagia), with a median suspension of 4 days (range 1–7). All patients underwent concomitant CDDP chemotherapy: 100 mg/mq triweekly schedule in 9 cases weekly 40 mg/mq in 15 patients. However, all patients received a total CDDP dose of at least 200 mg/mq with a dose intensity of 66.6% of a maximum hypothetical total dose of 300 mg/mq of CDDP. Main CRT features are summarized in Table 1. According to CTCAE v.4.01 “nausea” and “vomiting” items (Table 2), during treatment, the worst nausea grade reported by 11 patients (45.8%) was G0, while 8 patients (33.3%) reported G1, and 5 patients (20.8%) reported G2 nausea. Among patients, 18 (75%) reported the worst vomiting grade as G0, 5 patients (21%) reported G1, and only one patient (4%) experienced G2. G2 nausea was found to be more frequent in patients treated with the CDDP schedule q21 (4/5 cases), as same as G1 vomiting (4/5), even if the patient who reported G2 vomiting underwent weekly CDDP. The overall acute CR rate was 89% (95% CI 75%–97%). Mean absolute weight loss at the end of treatment was 5.9 kg (SD +/− 4) with the majority of patients (50%) reporting a loss extent equal or lower than 5% and 5 patients having a weight loss extent > 10% of the initial value. No correlation was found between nausea and DVC mean dose (*p* = 0.573), AP mean dose (*p* = 0.869) and weight loss (*p* = 0.348). FLIE scores were recorded at baseline, T1 (2 weeks) and T2 (5 weeks). The mean baseline value was 124 points (range 118–126), while mean scores reported at week 2 and week 5 were 105.5 and 97.5 points, respectively. The mean FLIE score decrease was 10.5 (baseline–T1), 5.5 (T1–T2) and 20.5 (baseline–T2) points (Fig. 2). Transposed on a 100-score scale, these corresponded to 83.7 and 77.3. The EORTC QLQ-HN 43 questionnaire was administered to all patients before treatment beginning (baseline), at week 2 (T1) and week 5 (T2). All patients completed the 43 items. The HN43 questionnaire analysis (Fig. 3) showed significantly higher scores of pain in the mouth (*p* < 0.001), swallowing (*p* < 0.001), teeth (*p* 0.037) domains at T2 assessment in comparison with baseline. Furthermore, worse T1 and T2 scores for senses (*p* < 0.001 and *p* < 0.001, respectively), social eating (*p* 0.001 and *p* < 0.001, respectively), open mouth (*p* 0.011 *p* < 0.001, respectively) and weight loss (*p* 0.006 and *p* < 0.001, respectively) domains (supplementary material 1) were collected. At univariate analysis (supplementary material 2), smoking 10–20 p/y and > 20 p/y (*p* 0.006 and *p* 0.027, respectively), ECOG PS 1 (*p* 0.038), IVa stage (*p* 0.010) were significantly related to worse pain in the mouth scores. Moreover, smoking 10–20 p/y negatively impacted on quality of life as reported in swallowing (*p* 0.004), dry mouth (*p* 0.050), senses (0.007), social eating (*p* 0.003) and open mouth (*p* 0.025) domains.

**Table 1:** Patients’ characteristics

Characteristic	No. of patients (%), *n* = 24
Median age, *y* (range)	64 (47–73)
Sex	
Male	19 (79.2%)
Female	5 (21.8%)
ECOG	
0	17 (70.8%)
1	7 (31.8%)
RT dose	
(IMRT SIB technique; 33 fractions) 66–69.9 Gy	24 (100%)
Cisplatin regimen	
40 mg/m^2^	15 (62.5%)
100 mg/m^2^	9 (37.5%)
Primary tumor site	
Oropharynx	14 (58.4%)
Oral cavity	3 (12.5%)
Larynx	3 (12.5%)
Nasopharynx	2 (8.3%)
Unknown primary	2 (8.3%)
AJCC staging	
≤ III	9 (37.5%)
IVA	12 (50%)
IVB	3 (12.5%)

ECOG, Eastern cooperative oncology group; AJCC, American joint committee on cancer 7th edition

**Table 2:** Acute treatment-related toxicity according to CTCAE version 4.01

	No. (%) by toxicity grade			
Toxicity (CTCAE v 4.01)	Grade 0	Grade 1	Grade 2	Grade 3
Nausea	8 (33.3%)	10 (41.7%)	6 (25%)	0
Vomiting	18 (75%)	5 (20.8%)	1 (4.2%)	0

## Discussion

Concerning the management of patients who undergo CRT, antiemetic treatment optimization is still necessary to effectively address CINV. Particularly this issue is underreported in head and neck cancer patients [20, 21] and main data derives from breast and lung cancer series [22–24]. In this regard, the impact of triple anti-HEC combination for HNSCC has been, so far, poorly investigated. New evidence suggests that NK1 RAs are effective in controlling CINV in certain types of cancer. However, this issue is underexplored in head and neck cancer patients. Only five prospective studies have investigated the efficacy and safety of NK1 RAs in the prophylaxis of CINV, which were focused on multiple-site tumors [25–31]. These studies showed that NK1 RAs are superior to other treatments in preventing nausea and vomiting, and are well tolerated. Nonetheless, it is unclear whether different types of tumors, chemotherapy regimens and radiation schedules would have similar results. This study investigated the rate of CINV in head and neck cancer patients undergoing prophylaxis with triple agents for CRT. We resumed the main data available from the literature in Table 3 [25, 27, 30–32].

**Table 3:** Main published evidence on CINV rate in head and neck cancer patients undergoing prophylaxis with triple agent for CRT

Trial (first author, ref)	*N*° pts	Study protocol	Cisplatin-RT (*n*)	Antiemetic regimen or NK1-RA tested	CR rate (no vomiting)	Nausea overall/significant nausea	Quality of life	Comments
Jahn et al. [24]	59 (36 HNC)	Prospective observational	all concurrent local RT-CDDP	I Group: (Control): *5-HT3-RA (onda or tropi) on the day of CDDP treatment and a steroid (dexa) on the day of CDDP treatment and for another 2 days* II Group (aprepitant): *5 HT3-RA* + *dexa (same regimen as in the control group)* + *aprepitant 125 mg day 1 and 80 mg the following days (another 2 days for single-day CDDP and another 6 days for the 5-day CDDP regimen)*	The CR rates for cycle 1 and 2 were 86.4% and 83.3% for the aprepitant group and 72.7% and 63.6% for the control group, respectively	Nausea was significantly more prominent in the control arm in comparison with the standard arm in cycle 1 and 2 (*p* = *.03)*	n.a	Although the primary endpoint was not obtained, the absolute difference of 10% in efficacy was reached, which is defined as clinically meaningful for patients by international guidelines groups
Navari et al. [26]	101 (51 group I;50 group II)	Phase III	all concurrent local RT-CDDP	I Group (OPD): *10 mg oral olanza, 0.25 mg IV palo, 20 mg IV dexa before cht on d1 and 10 mg/day of oral olanza before cht on d 2–4* II Group (FPD): *150 mg IV fosa, 0.25 mg IV palo, 12 mg IV dexa before cht on d1 and 4 mg dexa PO BID, before cht d 2–3*	ACUTE:I group (OPD) 84% vs II group (FPD) 7% (*p* = 0.61); DELAYED:I group (OPD) 84% vs II group (FPD) 74% (*p* = 0.46)	pts with no nausea: OPD: 86% acute, 71% delayed, FPD: 78% acute, 40% delayed, (? > .01 for acute; ? < .01 for delayed)	n.a	CR was similar for OPD and FPD; nausea in the delayed and overall periods was significantly improved with OPD compared with FPD (? < .01)
Wang et al. [29]	40	Phase II	CDDP (q21) with concurrent RT	*oral aprepitant 125 mg d1, then 80 mg on d2-5; onda 8 mg d1; and dexa 12 mg d1, then 8 mg on d2-5*	The overall CR rate was 86.0% (95% CI 72.1–94.7)	The emesis-free response and nausea-free response in overall phase were 88.4% (95% CI 74.9–96.1) and 60.5% (95% CI 44.4–75.0), respectively	analyzed but not yet reported ( FLIE, EORTC QLQ-C30 and QLQ-H&N35)	
D’souza et al. [30]	524	Phase III	Weekly cisplatin below 50 mg/m^2^ with concurrent RT	I Group (265): *Aprepitant, 5HT3 antagonist and dexa* II Group (256): *5HT3 antagonist and dexa*	I Group: no vomiting in 72.3%	Nausea: I Group 42.3% vs 47.9% overall (*p* = 0.010) Vomiting: I Group 26.8% vs 29.6% overall	n.a	Use of aprepitant significantly decreases nausea and is needed for weekly cisplatin regimens used in radiation in head and neck cancers
Stinson et al. [31]	199 (161 high-CDDP group)	Retrospective	High CDDP (≥ 50 mg/m^2^) or low CDDP (< 50 mg/m^2^) with concurrent RT	High CDDP Prophylaxis of acute CINV: *Onda 8 mg; dexa 8 mg; aprepitant 125 mg;* Prophylaxis of late CINV: *Onda 8 mg BID for 2 days; dexa 8 mg QD for 3 days; aprepitant 80 mg QD for 2 days; prochlorperazine 10 mg as needed*	High CDDP: 31%	nausea (not specified) 38.5%	n.a	CINV control for patients receiving high emetogenic chemotherapy was sub-optimal

RT, radiotherapy; NK1-RA, neurokinin1 receptor antagonist; CR, complete response; QoL, quality of life; HNC, head and neck cancer; CDDP, cisplatin; Fosa, fosaprepitant; Palo, palonsetron; Dexa, dexamethasone; n.a., not assessed; olanza, olanzapine; cht, chemotherapy; OPD, olanzapine–palonsetron–dexamethasone; FDP, fosaprepitant–palonsetron–dexamethasone; BID, bis in die; 5-HT3-RA, 5-hydroxytryptamine receptor antagonists; Onda, ondansetron; Tropi, (onda or tropi)

According to data of a recent phase III trial, the use of aprepitant significantly decreases nausea and should be adopted for weekly cisplatin regimens used in HNSCC RT. Indeed, after the comparison of two schedules for CINV control with or without aprepitant combined with 5HT3 antagonist and dexamethasone, D’souza et al. [31] reported a rate of nausea of 42.3% in the aprepitant group vs 47.9% in the control group. Stinson et al. [32] evaluated two strategies of prophylaxis of acute and late CINV in a retrospective cohort of HNSSC patients undergoing CRT collecting a nausea rate of 38.5%. They concluded that there is a need for better implementation of CINV control for patients receiving HEC. Wang et al. [30] conducted the first prospective trial to assess the efficacy and safety of aprepitant in combination with ondansetron and dexamethasone for preventing nausea and vomiting in HNSCC patients receiving triweekly CDDP CRT. The study’s primary endpoint was met, with an overall CR rate of 86.0%, indicating that the triple antiemetic regimen provided effective protection against CINV in patients with LA-HNSCC. Previous prospective studies on NK1RAs for CINV prophylaxis reported CR rates between 48 and 76% [25, 27]. However, both Wang’s [30] and our study’s results showed that the triple antiemetic regimen achieved excellent antiemetic efficacy for HEC regimen.

The amount of the literature pertaining to the role of FOS in HNSCC patients treated with CRT is limited and the optimal management of CINV in this setting of patients is currently under investigation.

Overall, in comparison with studies on NK1 RAs for CINV prophylaxis, our study utilized a triple antiemetic regimen consisting of FOS, ondansetron and dexamethasone, which showed a high efficacy in controlling CINV. However, the incidence of nausea in our study remained relatively high at 54.2%. A previous small randomized controlled trial indicated that the addition of olanzapine increased the control rate of nausea from 40 to 71% [27], which suggests that further research could be conducted to improve the residual nausea during the triple regimen.

Therefore, the contribution of RT in the occurrence of nausea and emesis in patients receiving RT with concomitant chemotherapy is not well documented. RINV is a critical and underreported complication of RT. Historically, it was suggested that patients undergoing CRT should be given antiemetic prophylaxis based on the type of chemotherapy used, unless the risk of RINV outweighed that of CINV [33]. Of note, the heterogeneity in RINV trials designs limited the consensus among the authors and the homogeneity of data leading to inadequacy in antiemetic recommendations. In this regard, McKenzie et al. [12] examined the MASCC/ESMO, ASCO and NCCN antiemetic guidelines in order to identify a common denominator. After assessing the strength and supporting evidence within the literature, the authors concluded that none of the RINV recommendations published reported a differentiation of risk level between nausea and vomiting or suggested specific management. The risk categories for RT have been defined mostly by the site of radiation. The study did not analyze the potential influence of other factors such as gender, age, previous alcohol use and previous experience of nausea and vomiting [20, 21, 34].

5HT3 RAs, which have demonstrated relevant benefits in the acute control of emesis, represent the standard treatment for RINV. Nonetheless, the effectiveness and safety of newly developed drugs used for preventing CINV should also be assessed for patients undergoing CRT. In the GAND-emesis trial [28], the authors explored the use of FOS in conjunction with palonosetron and dexamethasone as a preventive measure for nausea and vomiting in patients with cervical cancer undergoing 5 weeks of RT and concurrent CDDP. They observed that the FOS group had a significantly reduced risk of emesis in comparison with the group receiving palonosetron and dexamethasone alone, with a favorable tolerability profile. In the setting of HNSCC patients, experiences focused on the role of FOS in preventing RINV are still missing.

According to the above-mentioned guidelines, RT for HNSCC has been classified as a therapy with low risk of causing emesis. However, a number of publications suggest that IMRT—current state of the art—is related to higher rates of RINV [35]. It is known that nausea and vomiting may arise due to the incidental exposure of nontarget organs at risk to radiation during therapy. In this regard, the higher incidence of RINV is related to higher dose deposited on the brain stem for which is mandatory in clinical practice the dose constraint of 54 Gy [36] in order to avoid necrosis. However, there is still the unmet need for specific correlation between dose to brain stem and substructures, such as the AP and DVC, and the occurrence of RINV. The side effects of dysgeusia and nausea are commonly experienced by HNSCC patients treated with either exclusive RT or combined strategies [37, 38]. Indeed nausea may be significantly influenced by the occurrence of dysgeusia. In this regard, some authors have investigated the use of chemotherapy-induced taste alteration scale (CiTAS) to prospectively evaluate this adverse event [39]. Of note, a recovery for discomfort, phantogeusia–parageusia and general taste impairment at 6 months was observed by Martini and colleagues [39].

To the best of our knowledge, this is the first study to establish a relationship between the mean dose delivered to vomiting structures and the development of RINV. The use of NK1-RA may have a potential role in managing nausea and vomiting in head and neck cancer patients receiving IMRT. This is supported by a previous study where patients who received radiation dose to the DVC experienced a higher incidence of nausea and vomiting during head and neck IMRT [40]. Conversely, we did not find a correlation between nausea and DVC mean dose (*p* = 0.573) and AP mean dose (*p* = 0.869). However, this study does not provide a definitive conclusion regarding the role of an NK1-RA in radiation therapy.

Despite a good CR reported with triple antiemetic therapy in HNSCC, data emerged from FLIE analysis showed a lack of control in delayed nausea, which could be a crucial factor to better control RINV, particularly where nutritional status is an issue.

The present study has several limitations. Firstly, this is a pilot experience with a small sample size of patients, but considered sufficient for the purpose of the study. A larger sample would be useful to confirm the results obtained. Despite using an updated QoL questionnaire for assessments, we did not carry out VAS evaluations. Moreover, patients included in our cohort underwent two different regimens of CDDP administration, which could cause a bias taking into account the higher rate of vomiting with the 3-weekly schedule of CDDP [41]*.*

However, all patients were consistently treated, with a good CDDP cumulative dose of at least 200 mg/m^2^.

## Conclusions

Despite the persistence of delayed nausea, the incorporation of fosaprepitant into the ondansetron–dexamethasone regimen proved to be an effective measure against nausea and emesis in patients with LA-HNSCC who were receiving concomitant cisplatin-based chemotherapy and RT. No correlation has been found between nausea and median dose to DVC and AP.

Further experiences are needed to confirm the incidence, the pathophysiology of nausea and vomiting and the effect of combination of antiemetic regimen in patients with HNSCC. Moreover, to determine the possible role of fosaprepitant in the setting of chemoradiotherapy, randomized phase 3 studies are necessary.

### Supplementary Information

Below is the link to the electronic supplementary material.

Supplementary file1 (XLS 93 KB)

## Data Availability

Not applicable.
